# Local coronary endothelial dysfunction varies with the extent of coronary disease: a 3 T MRI study

**DOI:** 10.1186/1532-429X-11-S1-P132

**Published:** 2009-01-28

**Authors:** Allison Hays, Sebastian Kelle, Glenn A Hirsch, Gary Gerstenblith, Robert G Weiss, Matthias Stuber

**Affiliations:** Johns Hopkins, Baltimore, MD USA

**Keywords:** Flow Velocity Measurement, Isometric Handgrip, Coronary Endothelial Dysfunction, Maximum Grip Strength, Peak Diastolic Velocity

## Introduction

Endothelial-dependent coronary artery vasoreactivity is an important indicator of vascular function and predicts cardiovascular events [1]. Non-invasive measures of endothelial dysfunction are typically obtained in the brachial arteries, which are exposed to systemic risk factors but which rarely develop severe atherosclerosis or plaque rupture. Endothelial injury plays a critical, causal role in the development and progression of local atherosclerosis, a regionally heterogeneous process in the coronary arteries. We therefore posit that local endothelial function varies throughout the coronary tree in patients with disease and may contribute to local atherosclerosis. By means of previously described non-invasive 3 T MRI methods combined with isometric handgrip to assess endothelial-dependent coronary vasoreactivity [2], we therefore sought to test the hypothesis that local endothelial function varies within the coronary vasculature and is more deranged in regions with significant coronary atherosclerosis than in those with mild disease.

## Methods

Eleven patients (59 ± 6.2 years, mean ± SD, 3 women) with x-ray-defined coronary artery disease (CAD) were recruited and imaged using a 3 T MRI scanner (Achieva, Philips, Best, NL). In each patient, two arteries were imaged in cross-section: one artery (LAD or RCA) with severe stenosis (≥60%) and the contralateral artery with no significant stenosis (<30%) by x-ray angiography. Baseline imaging at rest for cross-sectional coronary artery area measurements was followed by coronary flow velocity-encoded MRI for flow velocity measurements. Alternating anatomical and velocity-encoded images were collected at baseline, and during 4 minutes of continuous isometric handgrip (at 30% of maximum grip strength). Three patients additionally received 0.4 mg of sublingual nitroglycerin and images were collected after five minutes. MRI parameters for anatomical and flow imaging respectively were: echo time (TE) = 1.5 ms/3.5 ms, radiofrequency (RF) excitation angle = 20° and spectral spatial excitation (both), breath-hold duration ~17–23 sec, acquisition window = 10 ms/27 ms, repetition time (TR) = 14 ms/34 ms, 21/11 spiral interleaves/cine frame, spatial resolution = 0.89 × 0.89 × 8.0 mm^3^/0.8 × 0.8 × 8 mm^3^ with velocity encoding = 35 cm/second. Blood pressure and heart rate were recorded at rest and during handgrip. I mages were analyzed for cross-sectional area changes (Cine version 3.15.17, General Electric, Milwaukee, WI) and for peak diastolic coronary flow velocity (FLOW Version3.0, Medis, NL). Coronary flow (mL/min) was calculated as: coronary cross-sectional area × coronary artery peak diastolic velocity × 30 [3].

## Results

**Figure 1:**
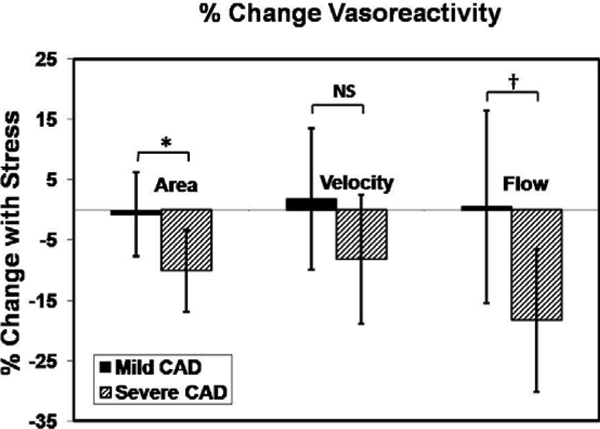
**Percent change (mean ± SD) from baseline in coronary artery area, peak diastolic coronary flow velocity and flow during isometric handgrip stress for arteries with mild vs. severe coronary disease**. (*p = 0.007, † p = 0.02 mild vs. severe CAD)

Nine patients had adequate image quality in both arteries for coronary area measurements and eight patients had sufficient image quality for flow velocity measurements. The mean percent increase in rate pressure product (heart rate*systolic blood pressure) with stress was 23 ± 10.7% (p < 0.001 vs. baseline). Isometric handgrip stress induced significant declines in cross-sectional area in arterial segments with severe coronary disease (baseline 15.8 ± 3.3 vs. stress 14.2 ± 2.7 mm^2^, p = 0.005). There was no significant change in coronary area with stress in the arteries with minimal disease (baseline 12.5 ± 2.8 vs. stress 12.3 ± 2.8 mm^2^, p = 0.58). All arterial segments dilated with nitroglycerin in the three patients that were studied. Although there was no significant change in peak diastolic coronary flow velocity with stress in both severe and mildly diseased arteries, there was a significant reduction in flow with stress in arteries with severe disease compared to the mildly diseased arteries (Figure 1).

## Conclusion

Using 3 T MRI combined with isometric handgrip exercise to quantify coronary endothelial-dependent vasoreactivity, we observe differences in local endothelial function throughout the coronary tree with more severe impairment of endothelial function in regions with more advanced coronary artery disease. This occurs despite an intact vasodilatory response to nitroglycerin. The present findings demonstrate local differences in coronary endothelial function related to disease severity and raise concerns about the ability of a single measure of endothelial function in a peripheral vessel to accurately reflect the spectrum of endothelial function present in diseased coronary arteries. This novel noninvasive approach may offer important insights into the pathobiology of atherosclerosis, the local progression of CAD and the identification of high risk lesions.
